# Discovery of Volatile Biomarkers for Bladder Cancer Detection and Staging through Urine Metabolomics

**DOI:** 10.3390/metabo11040199

**Published:** 2021-03-26

**Authors:** Joana Pinto, Ângela Carapito, Filipa Amaro, Ana Rita Lima, Carina Carvalho-Maia, Maria Conceição Martins, Carmen Jerónimo, Rui Henrique, Maria de Lourdes Bastos, Paula Guedes de Pinho

**Affiliations:** 1 UCIBIO/REQUIMTE, Department of Biological Sciences, Laboratory of Toxicology, Faculty of Pharmacy, University of Porto, 4050-313 Porto, Portugal; famaro@ff.up.pt (F.A.); ritacmlima@hotmail.com (A.R.L.); mlbastos@ff.up.pt (M.d.L.B.); 2 Cancer Biology & Epigenetics Group–Research Centre, Portuguese Oncology Institute of Porto (CI-IPOP), 4200-072 Porto, Portugal; carina.carvalho.maia@ipoporto.min-saude.pt (C.C.-M.); mconceicaom@ipoporto.min-saude.pt (M.C.M.); carmenjeronimo@ipoporto.min-saude.pt (C.J.); rmhenrique@icbas.up.pt (R.H.); 3 Department of Pathology, Portuguese Oncology Institute of Porto, 4200-072 Porto, Portugal; 4 Department of Pathology and Molecular Immunology, Institute of Biomedical Sciences Abel Salazar–University of Porto (ICBAS-UP), 4050-313 Porto, Portugal

**Keywords:** bladder cancer, volatile biomarkers, diagnosis, staging, urine, gas chromatography-mass spectrometry

## Abstract

Timely diagnosis is crucial to improve the long-term survival of bladder cancer (BC) patients. The discovery of new BC biomarkers based in urine analysis is very attractive because this biofluid is in direct contact with the inner bladder layer, in which most of the neoplasms develop, and is non-invasively collected. Hence, this work aimed to unveil alterations in the urinary volatile profile of patients diagnosed with BC compared with cancer-free individuals, as well as differences among patients diagnosed at different tumor stages, to identify candidate biomarkers for non-invasive BC diagnosis and staging. Urine analysis was performed by headspace solid-phase microextraction coupled with gas chromatography-mass spectrometry (HS-SPME-GC-MS). The results unveiled that BC patients have a distinct urinary volatile profile characterized by higher levels of several alkanes and aromatic compounds, and lower levels of aldehydes, ketones and monoterpenes. Seventeen significantly altered volatiles were used to evaluate the performance for overall BC detection, disclosing 70% sensitivity, 89% specificity and 80% accuracy. Moreover, distinct urinary volatile profiles were found among patients diagnosed at different tumor stages (Ta/Tis, T1 and ≥T2). This work identified distinct urinary volatile signatures of BC patients with potential for non-invasive detection and staging of bladder cancer.

## 1. Introduction

Bladder cancer (BC) is the 10th most common form of cancer worldwide and one of the most lethal [1]. It is typically diagnosed in people aged over 65 years and the incidence rates differ among men and women, with a four times higher incidence in men [2]. The main risk factors associated with BC development are smoking, aging and occupational exposure [3]. BC can be categorized into non-muscle invasive (NMIBC) and muscle invasive (MIBC) tumors. NMIBC is restricted to the urothelium [stage Ta/Tis, as defined by the tumor, node, metastasis (TNM) classification system] or lamina propria (stage T1) accounting for 70–80% of all cases, whereas MIBC (stage ≥ T2) invade the muscularis propria or beyond [2]. This distinction is very important since NMIBC patients are mostly treated with bladder-sparing strategies, including trans-urethral resection, complemented with intravesical immunotherapy or chemotherapy, whereas MIBC patients are treated with radical cystectomy plus neoadjuvant or adjuvant chemotherapy or, eventually, immunotherapy [4].

Hematuria (gross or microscopic) is the most common clinical manifestation of BC, occurring in 80–90% of patients [4]. Current standard diagnostic methods include cystoscopy, urinary cytology and imaging techniques (computed tomography (CT) and magnetic resonance imaging (MRI)) [5]. Cystoscopy is an invasive procedure which directly visualizes the bladder lining for presence of a tumor, with overall reported sensitivity and specificity of 68–100% and 57–97%, respectively [6]. This procedure can lead to urethral injury, urinary tract infection and hematuria. Urinary cytology, which detects cancer cells in urine, is a non-invasive method particularly useful as a complement to cystoscopy, with sensitivity and specificity for BC detection ranging 13–86% and 73–100%, respectively [6]. Besides the very low sensitivity for detection of low-grade tumors, this technique is operator dependent and can be hindered by low cellular yield, urinary tract infection and other comorbidities [5]. CT and MRI are mainly used for staging and evaluation for distant metastases, but concern remains regarding their ability to detect flat urothelial lesions, and accurately perform local-staging and nodal detection [7]. 

Urinary biomarkers represent an attractive alternative or adjunct to current BC standard diagnosis. An ideal BC biomarker should be non-invasive, objective, easy to interpret and have high sensitivity and specificity [8]. There are some urine tests currently approved by Food and Drug Administration (FDA) based on altered proteins [e.g., nuclear matrix protein 22 (NMP-22), complement factor H-related protein (BTA TRACK)] or chromosomal aberrations (e.g., UroVysionTM) [6,9]. However, these tests have not replaced the current standard diagnostic methods as they are affected by hematuria, infection or inflammation leading to false-positive results [9]. 

In the era of precision medicine, multiple levels of molecular profiling (e.g., genome, epigenome, transcriptome, proteome, metabolome) of BC have been investigated for accurate diagnosis, prognostication, and prediction of treatment response [9]. In particular, the volatile fraction of the urine metabolome has shown very promising results for diagnosis of prostate and gastrointestinal cancers, as recently reviewed [10]. However, only one study has explored the urinary volatilome for identification of potential biomarkers for BC [11]. This study reported 89% sensitivity and 66% specificity, with overall 83% accuracy and area under the curve (AUC) of 0.868, for discrimination of BC (*n* = 72) from cancer-free controls (*n* = 46) based on the alterations in the levels of several ketones, aldehydes, acids and monoterpenes. 

Herein, we aimed to investigate the performance of urinary volatilome in discriminating patients diagnosed with BC from cancer-free controls, based on the analysis of urine by headspace solid-phase microextraction coupled with gas chromatography-mass spectrometry (HS-SPME-GC-MS). Because BC is more often diagnosed in men than women, urine samples from BC and control groups were gender matched. An age-match was not possible but the effect of this potential confounding factor on the levels of discriminant volatile metabolites was further investigated through correlation analysis. Moreover, volatilome alterations occurring among different tumor stages (Ta/Tis, T1 and ≥T2) were also investigated. 

## 2. Results

### 2.1. Differences in Urinary Volatile Profile of BC Patients vs. Controls

In this study, the performance of the urinary volatile profile for discrimination of BC (urothelial carcinoma) patients (*n* = 53) from cancer-free controls (*n* = 56) was evaluated. Most of the BC patients were diagnosed with NMIBC (Table 1), comprising stage Ta/Tis (*n* = 26) and T1 (*n* = 17), and the remainder 19% were diagnosed with MIBC, including stages T2 (*n* = 4), T3 (*n* = 4) and T4 (*n* = 2). 

**Figure 1:**
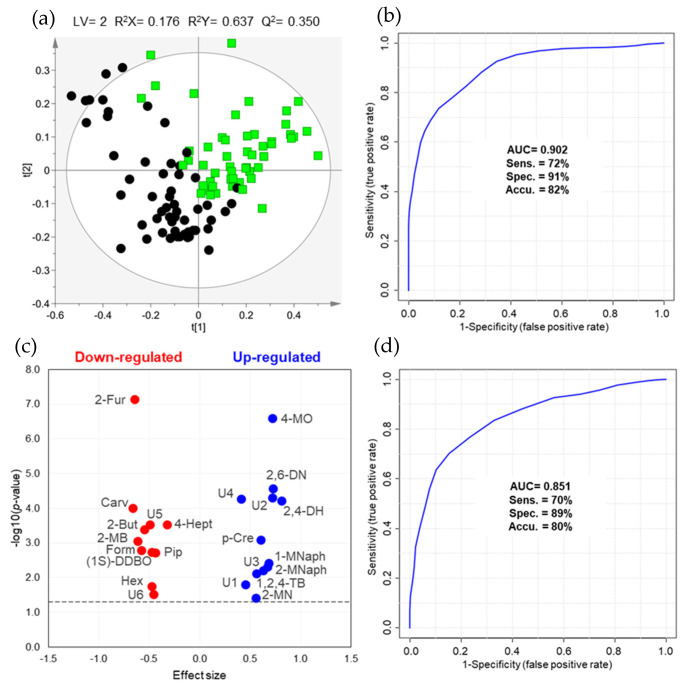
(**a**) PLS-DA scores scatter plot and (**b**) receiver operating characteristic (ROC) curve obtained for urinary volatile profile (VOCs and VCCs concatenated matrix), after variable selection (109 observations × 4184 variables), of BC patients (green squares, *n* = 53) and cancer-free controls (black circles, *n* = 56). (**c**) Volcano plot representing the set of twenty-three urinary volatile metabolites changing between BC patients and cancer-free controls. (**d**) ROC curve obtained for the set of seventeen discriminant volatiles (109 observations × 17 metabolites) that were formally (L1) and putatively (L2) identified. Metabolite abbreviations: 1-MNaph, 1-methylnaphthalene; (1S)-DDBO, (1S)-1,5-dimethyl-6,8-dioxabicyclo[3.2.1] octane; 1,2,4-TB, 1,2,4-trimethylbenzene; 2-But, 2-butanone; 2-Fur, 2-furaldehyde; 2-MB, 2-methylbutanal; 2-MN, 2-methylnonane; 2-MNaph, 2-methylnaphthalene; 2,4-DH, 2,4-dimethylheptane; 2,6-DN, 2,6-dimethylnonane; 4-Hept, 4-heptanone; 4-MO, 4-methyloctane; Carv, carvone; Form, formaldehyde; Hex, hexanal; Pip, piperitone; *p*-Cre, *p*-cresol (4-methylphenol); U1-6, unknown 1-6.

The combination of two different sample preparation and extraction protocols for volatile profiling of urine enabled the detection of a total of 220 chromatographic peaks [120 in volatile organic compounds (VOCs) protocol and 100 in volatile carbonyl compounds (VCCs) protocol]. The analytical precision was first checked through the projection of the quality control (QC) samples (*n* = 24), acquired intermittently throughout the analytical experiment, and of all urine samples under study in a principal component analysis (PCA) model (Figure S1). The tighter clustering of the QC samples revealed that the data was not affected by technical variation and presented good quality for multivariate analysis. The partial least squares-discriminant analysis (PLS-DA) model obtained for the urinary volatile profile of BC patients and controls after variable selection (109 observations × 4184 variables) is shown in Figure 1a. The scores scatter plot depicted a satisfactory separation between groups with a moderate predictive ability (*Q^2^* = 0.350, Figure S2), high AUC (0.902) and 72% sensitivity, 91% specificity and 82% accuracy (Figure 1b). 

The volatile metabolites unveiling variable importance to the projection (VIP)> 1 and statistically different levels between BC and controls are shown in the volcano plot (Figure 1c). Eleven metabolites were found down-regulated in BC, including aldehydes (2-furaldehyde, 2-methylbutanal, formaldehyde and hexanal), ketones (2-butanone and 4-heptanone), terpenoids (carvone and piperitone), one heterocyclic compound ((1S,5R)-1,5-dimethyl-6,8-dioxabicyclo [3.2.1]octane) and two unknowns, whereas twelve were up-regulated, including alkanes (2-methylnonane, 2,4-dimethylheptane, 2,6-dimethylnonane), aromatic compounds (1-methylnaphthalene, 2-methylnaphthalene, 1,2,4-trimethylbenzene and *p*-cresol) and four unknowns (Table 2, Table S1). From these, 2-furaldehyde and 4-methyloctane disclosed the highest AUC (0.800 and 0.787, respectively) and statistical significance (*p*-value < 0.0001 after FDR correction). 

Considering the set of seventeen discriminant compounds formally or putatively identified, i.e., volatiles denoted as L1 and L2 in Table 2 and excluding the six unknowns, the classification performance of BC vs. controls (Figure 1d) was similar to that of the matrix used to construct the PLS-DA model, with 70% sensitivity, 89% specificity and 80% accuracy, notwithstanding the lower AUC (0.851). Regarding the performance of this set to detect each tumor stage (Table 3), very high sensitivity (94%) and AUC (0.910) were found for stage T1, whereas low sensitivity was found for stage Ta/Tis (65%) and ≥T2 (60%), despite high specificity (84% for stage Ta/Tis and 91% for ≥T2).

Finally, the potential influence of age on the levels of the twenty-three statistically different metabolites was investigated through correlation (Table S2). As very low correlation coefficients (|*r*| ≤ 0.3) were found for all metabolites, it may be concluded that age differences did not significantly contribute for the metabolic alterations detected between BC and controls.

### 2.2. The Impact of NMIBC and MIBC on Urinary Volatile Profile

Concerning a possible distinction of the two NMIBC stages based on the urinary volatilome, the PLS-DA model of stage T1 vs. Ta/Tis (43 observations × 495 variables, Figure S3) unveiled some differences, with an AUC of 0.837, 71% sensitivity, 73% specificity and 72% accuracy. Patients diagnosed with stage T1 showed higher urinary levels of decane, octanal and one unknown metabolite (unknown 7), and lower levels of levomenthol and another unknown (unknown 8) (Table S3). However, none of these metabolites retained statistically significant differences after FDR correction.

**Figure 2:**
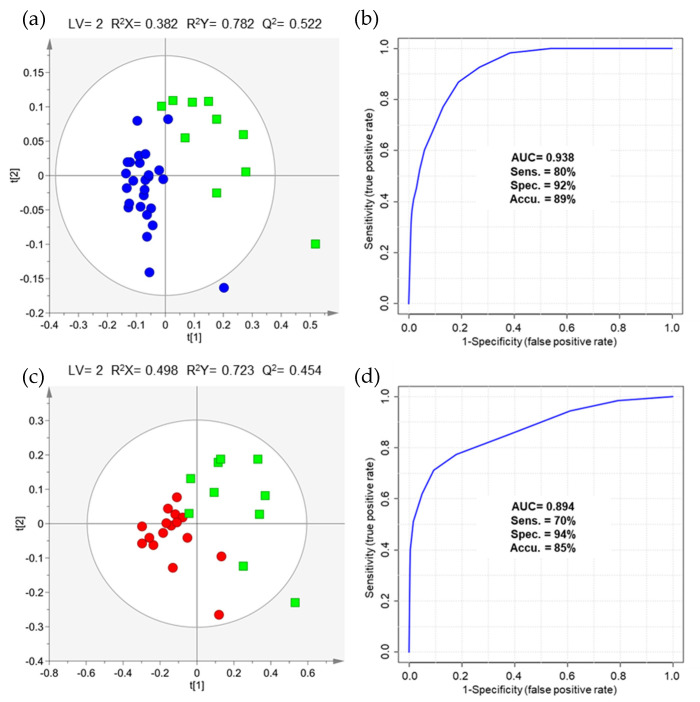
(**a**) PLS-DA scores scatter plot and (**b**) ROC curve obtained for urinary volatile profile (VOCs and VCCs concatenated matrix), after variable selection (36 observations × 486 variables), of patients diagnosed with MIBC (stages ≥ T2, green squares, *n* = 10) and NMIBC (stage Ta/Tis, blue circles, *n* = 26). (**c**) PLS-DA scores scatter plot and (**d**) ROC curve obtained for urinary volatile profile (VOCs and VCCs concatenated matrix), after variable selection (27 observations × 856 variables), of patients diagnosed with MIBC (stages ≥ T2, green squares, *n* = 10) and NMIBC (stage T1, red circles, *n* = 17).

To evaluate the differences between MIBC vs. NMIBC, two comparisons were performed after variable selection, namely stages ≥T2 vs. stage Ta/Tis (36 observations × 486 variables, Figure 2a) and stages ≥T2 vs. stage T1 (27 observations × 856 variables, Figure 2c), due to the discrepancy in the sample size of NMIBC and MIBC cohorts. Similar classification performances were found for both comparisons with AUC of 0.938, 80% sensitivity, 92% specificity and 89% accuracy for distinction of stages ≥T2 from stage Ta/Tis (Figure 2b), and AUC of 0.894, 70% sensitivity, 94% specificity and 85% accuracy for distinction of stages ≥T2 from stage T1 (Figure 2d).

**Figure 3:**
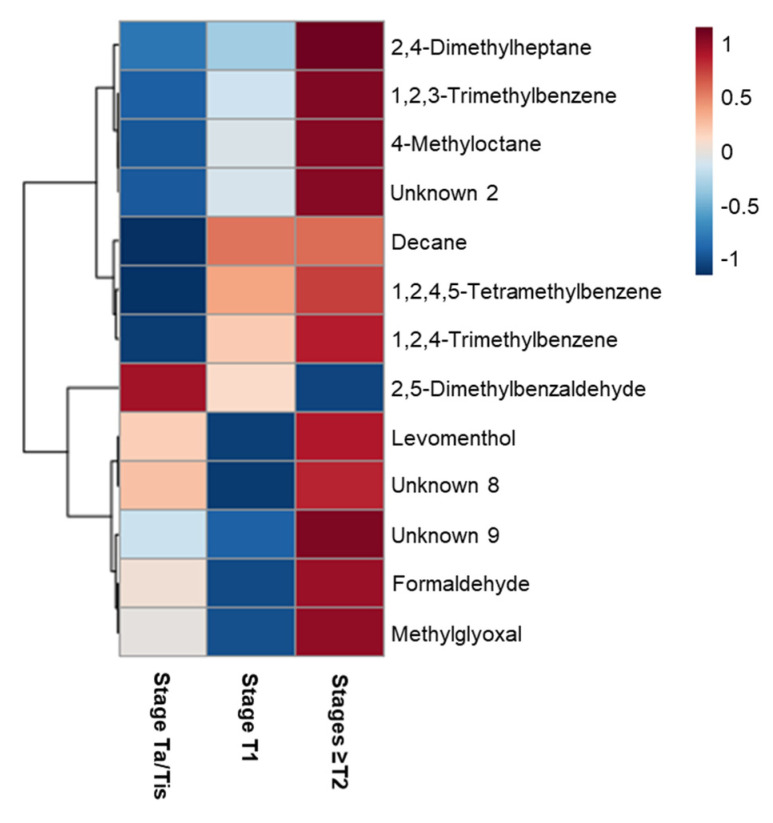
Heatmap illustrating the levels of urinary volatile metabolites changing between NMIBC (stage Ta/Tis and T1) and MIBC (stages ≥T2) patients. Columns correspond to each sample group, while rows correspond to the mean normalized peak area of each discriminant volatile metabolite colored from minimum (−1, dark blue) to maximum (1, dark red).

The heatmap depicted in Figure 3 represents the urinary metabolites differing between MIBC and the two NMIBC groups, which are listed in detail in Tables S4 and S5. These results unveiled that urines of patients diagnosed with MIBC were characterized by higher levels of alkanes (2,4-dimethylheptane, 4-methyloctane and decane), aldehydes (formaldehyde and methylglyoxal), aromatic compounds (1,2,3-trimethylbenzene, 1,2,4-trimethylbenzene and 1,2,4,5-tetramethylbenzene) and two unknowns (unknown 8 and 9), and lower levels of 2,5-dimethylbenzaldehyde. These findings suggest that NMIBC and MIBC have distinct urinary volatile profiles, requiring further investigation in larger cohorts.

## 3. Discussion

The investigation of candidate volatile biomarkers for urological cancers has become of great interest over the last years due to a study by Willis et al. which showed that dogs were able to detect BC in urine samples by sniffing [22]. Based on this observation, several researchers have focused on the application of electronic noses, i.e., devices that mimic the human olfactory system, for analysis of volatile metabolites present in urine headspace of cancer patients and cancer-free individuals [23]. Studies performed in urine of BC patients have demonstrated that electronic noses using different technologies can detect BC with sensitivity ranging from 60 to 93% and specificity from 67 to 93% [24,25,26]. However, electronic noses are based on the differentiation of odor fingerprints instead of chemical composition, not allowing for the identification of specific biomarkers. Thus, we aimed to identify volatile biomarkers of BC based on the analysis of urine headspace by HS-SPME-GC-MS. Our results unveiled a set of seventeen discriminant volatiles, including several alkanes, aldehydes, aromatic hydrocarbons, heterocyclic compounds, ketones and monoterpenes, able to detect BC with an AUC of 0.851, 70% sensitivity, 89% specificity and 80% accuracy. Comparing with FDA-approved urinary biomarkers, this set of discriminant volatiles revealed better overall sensitivity and specificity than NMP-22 (69% and 77%, respectively) and BTA TRACK (65% and 74%, respectively) [27]. In contrast with these FDA-approved biomarkers, the set of seventeen volatiles did not show an increase in sensitivity with higher tumor stage, unveiling higher sensitivity for detection of stage T1 (94% sensitivity and 80% sensitivity). This fact may be explained by the distinct urinary volatile profiles found between NMIBC stages (Ta/Tis vs. T1) and NMIBC vs. MIBC (stages ≥T2), with the latter characterized by higher levels of several alkanes (2,4-dimethylheptane, 4-methyloctane and decane) and aromatic compounds (1,2,3-trimethylbenzene, 1,2,4-trimethylbenzene and 1,2,4,5-tetramethylbenzene), and lower levels of 2,5-dimethylbenzaldehyde.

The altered volatile metabolites may also provide new insights into the metabolic dysregulations occurring in BC, as suggested in Table 3 and Tables S3–S5. Several branched alkanes were found increased in urine of BC patients, namely 2-methylnonane, 2,4-dimethylheptane, 2,6-dimethylnonane and 4-methyloctane. The potential endogenous origin of branched alkanes has been attributed by some researchers to peroxidation of polyunsaturated fatty acids [28], although this hypothesis has been challenged by the fact that there are no branched polyunsaturated fatty acids in the human body [29]. Despite the lack of information regarding the biochemical pathways, 2,4-dimethylheptane and 4-methyloctane were found increased in the headspace of a human non-small cell lung cancer cell culture suggesting a cellular origin [30].

Four aldehydes (2-furaldehyde, 2-methylbutanal, formaldehyde and hexanal) were found statistically decreased in urine of BC patients. The biological origin of 2-furaldehyde is still unknown. The decrease in hexanal and 2-methylbutanal levels may be attributed to oxidation by aldehyde dehydrogenase (ALDH) to yield carboxylic acids or a decrease in aldehyde formation through lipid peroxidation [14]. Hexanal has also been found decreased in urine of patients diagnosed with other cancer types (e.g., colorectal cancer, prostate cancer, leukemia and lymphoma) [31,32,33,34,35,36,37,38,39], whereas 2-methylbutanal was found decreased in patients diagnosed with head and neck cancer [37]. The presence of formaldehyde in human body can be associated with several sources, such as folate derivatives breakdown, protein and nucleic acid demethylation, glycine and serine metabolism or exogenous sources (e.g., smoking and diet) [13]. In contrast with our study, formaldehyde was found elevated in the headspace of urines collected from patients diagnosed with BC and prostate cancer in another study [40].

Aromatic compounds, such as 1-methylnaphthalene, 2-methylnaphthalene, 1,2,4-trimethylbenzene and *p*-cresol, were found in significantly higher levels in urine of BC patients. Methylnaphthalenes may have an exogenous source (e.g., cigarette smoke) [41]. The reactive metabolites generated in vivo from the metabolization of these molecules may damage body organs by modification of proteins [14,41]. *p*-Cresol (4-methylphenol) may be associated with a dysregulation in tyrosine and phenylalanine metabolism [15] and has been found increased in urine of patients diagnosed with lung, colorectal and breast cancers, as well as leukemia and lymphoma [36,38,42].

The presence of ketones in human body may be related to β-oxidation of fatty acids [12]. The decrease in 4-heptanone levels corroborate a previous study performed in urine of BC patients [11]. Moreover, 2-butanone and 4-heptanone have been found altered in other cancer types but the direction of the variation differed among studies [31,32,37,39,43,44,45,46]. Finally, lower levels of two monoterpenes (carvone and piperitone) were observed in urine of BC patients. Terpenes are present in herbal and dietary plants and can participate in lipid and carbohydrate metabolisms [16,17]. Interestingly, decreased levels of piperitone in urine of BC patients were also previously reported [11].

Overall, our study unveiled the presence of distinct volatile profiles in urine of BC patients with potential for tumor detection and staging. Future studies should include validation of these results in a larger cohort of samples, and improvement of sensitivity and specificity rates for overall BC detection as well as better diagnosis of NMIBC cases. In addition, it is very important to consider other concomitant conditions (e.g., hematuria, urinary tract infection) that may influence the urinary volatilome. Furthermore, the simultaneous analysis of urine by GC-MS and electronic sensors may offer new insights into chemical composition of odor signatures that can boost urinary volatilome analysis to clinical practice.

## 4. Materials and Methods

### 4.1. Chemicals

2-Butanone (≥99%), 2-furaldehyde (≥99%), 2-methylbutanal (90%), 4-heptanone (97%), carvone (≥98.5%), decanal (95%), decane (≥99%), formaldehyde (37% aqueous solution), hexanal (97%), methylglyoxal (40% aqueous solution), O-(2,3,4,5,6-pentafluorobenzyl)hydroxylamine hydrochloride (PFBHA) (≥ 99%), octanal (≥99%) and *p*-cresol (4-methylphenol) (≥98%) were purchased from Sigma-Aldrich (Madrid, Spain). Sodium chloride was supplied by VWR (Leuven, Belgium).

### 4.2. Patients and Sample Collection

A total of 109 subjects were enrolled in this study, including 53 patients diagnosed with BC (urothelial carcinoma) and 56 cancer-free individuals (control group). Urine samples were collected at the Portuguese Oncology Institute of Porto (IPO Porto), upon Ethics Committee approval (Ref. CESIPOFG-EPE:019/08), and in accordance with the principles included in the Declaration of Helsinki. All subjects signed informed consents before entering the study. The demographic and clinical data of BC patients and controls are depicted in Table 1.

Voided urine was collected from all participants in early morning (non-fasting). After collection, urine samples were centrifuged (3076 *g*, 20 min, 4 °C) and the supernatant was stored at −80 °C until analysis. QC samples, comprising a pool of 10 control urines, were prepared and stored at −80 °C (divided into aliquots), to evaluate technical precision.

### 4.3. Sample Preparation

Urine samples were prepared according to two protocols previously developed by our group [31]. Briefly, the first protocol was focused on the analysis of VOCs in general, consisting in the addition of NaCl (0.54 g) to urine (2 mL) in a glass vial (10 mL). The second protocol, developed to analyze the VCCs, consisted in the mix of O-(2,3,4,5,6-pentafluorobenzyl)hydroxylamine hydrochloride (PFBHA) (7.5 µL, 40 g/L solution) with urine (250 µL) in a glass vial (10 mL). VOCs and VCCs extraction were then performed by headspace solid-phase microextraction (HS-SPME). In VOCs protocol, the sample was first incubated (11 min, 44 °C, 250 rpm), followed by compound extraction from headspace (30 min, 44 °C, 250 rpm) using a 50/30 μm divinylbenzene/carboxen/polydimethylsiloxane (DVB/CAR/PDMS) fiber (Supelco Inc., Bellefonte, PA, USA), and thermal desorption of VOCs into the GC system (4 min, 250 °C), performed in a Combi-PAL autosampler (Varian Pal Autosampler, Zwingen, Switzerland). In VCCs protocol, the sample was first incubated (6 min, 62 °C, 250 rpm), followed by volatiles extraction from headspace (51 min, 62 °C, 250 rpm) using a polydimethylsiloxane/divinylbenzene (PDMS/DVB) fiber (Supelco Inc., Bellefonte, PA, USA), and thermal desorption into the GC injector (5 min, 250 °C), performed in Bruker CTC PAL-xt autosampler (Bruker Daltonics, Bremen, Germany).

### 4.4. GC-MS Analysis and Metabolite Identification

A 436-GC model coupled to a SCION single quadrupole (SQ) mass spectrometer (Bruker Daltonics, Bremen, Germany) was used for VOCs analysis. The GC system was equipped with a fused silica capillary column (Rxi-5Sil MS, 30 m × 0.25 mm internal diameter × 0.25 μm; Restek Corporation, U.S., Bellefonte, PA, USA) and high purity helium C-60 (Gasin, Porto, Portugal) was used as carrier gas at a constant flow rate of 1.0 mL/min. The oven temperature was programmed at 40 °C for 1 min, increased at 5.0 °C/min to 250 °C where it was held for 5 min, and then increased at 5.0 °C/min to 300 °C. The injector, transfer line, ion source and manifold temperatures were maintained at 250, 250, 260 and 41 °C, respectively. The SQ-MS was operated in electron impact (EI) mode (70 eV). Data acquisition was performed in full scan mode from *m/z* 40 to 400 with a scan time of 500 ms. For VCCs analysis, a 436-GC model coupled to an EVOQ triple quadrupole (TQ) mass spectrometer (Bruker Daltonics, Bremen, Germany) was used. The column and the carrier gas were the same described above for VOCs analysis. The oven temperature was programmed at 40 °C for 1 min, increased at 5.0 °C/min to 250 °C where it was held for 5 min, and then increased at 20.0 °C/min to 300 °C. The injector, transfer line, ion source and manifold temperatures were maintained at 250, 260, 270 and 41 °C, respectively. The TQ-MS was operated in EI mode (70 eV) and data acquisition was performed in full scan mode from *m/z* 35 to 600 with a scan time of 250 ms. The software used in the two equipment was a Bruker Daltonics MS workstation (version 8.2.1, Bruker Daltonics, Bremen, Germany). Urine samples were randomly injected, and QCs were repeatedly analyzed on every five samples.

Metabolite identification was performed by comparing the mass spectra and retention indices (RI), determined using a commercial hydrocarbon mixture (C6–C20), obtained for urine samples with the National Institute of Standards and Technology (NIST) standard reference database (version 14, Gaithersburg, MD, USA). A tolerance of ± 20 was accepted between the experimental and NIST RI and the minimum reverse match considered was 770. The identification was further confirmed by analysis of commercial standard compounds under the same conditions. This procedure enabled the definition of levels of confidence in metabolite identification as recommended for metabolomic studies [20,21].

### 4.5. Data Pre-Processing and Statistical Analyses

GC-MS chromatograms were pre-processed in MZmine-2.52 [47] after conversion to netCDF. The same pre-processing step were applied for VOCs and VCCs data, including crop filtering, peak detection, chromatogram builder, deconvolution and alignment with slightly differences in parameters as listed in Table S6. After pre-processing, artefact peaks from the fiber and chromatographic column (e.g., siloxanes and cyclosiloxanes) were manually removed from the matrices. A whole matrix was constructed by concatenating VOCs and VCCs matrices (133 observations × 10,709 variables), followed by total area normalization. Before multivariate analysis, a variable selection method was applied to the concatenated matrix based on the unpaired Mann-Whitney U test, performed in Metaboanalyst 4.0 [17]. Therefore, a final matrix containing only the variables with *p*-value < 0.05 was scaled to pareto and used for multivariate analysis. PCA, PLS-DA, seven-fold cross-validation and permutation test were performed using the final matrix for BC patients vs. cancer-free controls (109 observations × 4184 variables), stage T1 vs. Ta/Tis (43 observations × 495 variables), stage ≥ T2 vs. Ta/Tis (36 observations × 486 variables) and stage ≥ T2 vs. T1 (27 observations × 856 variables) in SIMCA-P 15 (Umetrics, Sweden). The metabolites considered important for group discrimination presented a VIP > 1 and *p*-value < 0.05. The choice of a univariate test for variable selection with respect to other more conventional approaches, such as PLS-VIPs or PLS-weights, relied on the degree of group separation and the predictive ability of the PLS-DA models generated, which were better when the univariate test was used. One limitation of this approach is the fact that it does not account for intercorrelations between metabolites that may reflect proximity in metabolic pathways. Ideally, the PLS-DA models would be validated independently on a test set inside the cross-validation procedure, but it was not possible due to the small sample cohort.

Receiver operating characteristic (ROC) curves were computed for each PLS-DA model after variable selection and the set of discriminant compounds formally (L1) and putatively (L2) identified, along with the AUC and confusion matrix (Metaboanalyst 4.0 [17]). Correction for multiple comparisons was performed using the false discovery rate (FDR) method [19] in R 3.5.3 software. Effect size [18], percentage of variation and AUC were also computed for each statistically significant compound. Finally, the correlation coefficients between age and the set of statistically significant compounds found altered between BC and the control group were computed in R 3.5.3 software, to investigate a potential influence of this confounding factor.

## 5. Conclusions

Our work demonstrated that BC patients have a distinct urinary volatile signature compared with cancer-free individuals, comprising higher levels of several alkanes and aromatic compounds, and lower levels of aldehydes, ketones and monoterpenes. This signature was able to detect BC with 70% sensitivity, 89% specificity, 80% accuracy and an AUC of 0.851. Regarding the detection of earlier BC stages, a remarkably high performance was found for stage T1 with 94% sensitivity, 80% specificity, 84% accuracy and AUC of 0.910, whereas stage Ta/Tis was less effectively detected with 65% sensitivity, 84% specificity, 78% accuracy and an AUC of 0.761. Surprisingly, the later stages of BC were not detected with higher sensitivity (60%), unveiling only better specificity (91%) and accuracy (86%). This may be explained by the distinct volatile profiles found between NMIBC stages (Ta/Tis vs. T1) and NMIBC vs. MIBC (stages ≥T2), with the latter characterized by higher levels of several alkanes and aromatic compounds, and lower levels of one aldehyde. These results emphasize that urinary volatile biomarkers are promising candidates for improving BC detection and staging. Indeed, the set of seventeen discriminant volatiles unveiled better overall sensitivity and specificity than some FDA-approved urinary biomarkers (NMP-22 and BTA TRACK).

Future studies should entail a larger cohort of samples, ideally matched for other confounding factors (e.g., age, smoking habits, lifestyle) and comprising other populations in which BC incidence is high (e.g., Western Europe, North America). Moreover, it will be important to consider other concomitant conditions that may influence the urinary volatilome, such as hematuria and urinary tract infection.

## Figures and Tables

**Table 1:** Demographic and clinical data of cancer-free individuals and BC patients.

Group	Number of Samples (F/M)	Age Range (years)	Mean Age ± SD (years)
Cancer-Free Controls	56 (16/40)	45–66	51.9 ± 5.2
BC Patients	53 (14/39)	43–87	68.9 ± 10.6
Ta/Tis	26 (6/20)	43–87	68.6 ± 10.8
T1	17 (6/11)	53–83	72.0 ± 8.9
T2	4 (1/3)	51–69	62.0 ± 7.5
T3	4 (1/3)	43–80	65.3 ± 15.0
T4	2 (0/2)	65–70	67.5 ± 2.5

F: female; M: male.

**Table 2:** List of twenty-three volatile metabolites found altered in urine of BC patients (*n* = 53) compared with cancer-free controls (*n* = 56).

Metabolite *^a^*	Effect Size ± ES_SE_ *^b^*	Variation ± Uncertainty (%)	*p*-Value Original	*p*-Value FDR *^c^*	AUC	Down- or Up-Regulated	HMDB ID	Potential Biochemical Pathway
***Alkanes***								
2-Methylnonane *^d, L2^*	0.56 ± 0.38	55.6 ± 15.4	0.0390	0.0477	0.615	↑	−	−
2,4-Dimethylheptane *^d, L2^*	0.81 ± 0.39	183.2 ± 23.6	<0.0001	0.0002	0.724	↑	−	−
2,6-Dimethylnonane *^d, L2^*	0.73 ± 0.39	122.1 ± 20.7	<0.0001	0.0002	0.734	↑	−	−
4-Methyloctane *^d, L2^*	0.72 ± 0.39	420.9 ± 37.7	<0.0001	<0.0001	0.787	↑	−	−
***Aldehydes***								
2-Furaldehyde (furan-2-carbaldehyde) *^e, L1^*	−0.65 ± 0.39	−48.4 ± 18.8	<0.0001	<0.0001	0.800	↓	HMDB0032914	-
2-Methylbutanal *^d, L1^*	−0.61 ± 0.38	−40.3 ± 15.4	0.0009	0.0021	0.685	↓	HMDB0031526	Aldehyde oxidation (ALDH) and lipid peroxidation [12]
Formaldehyde *^e, L1^*	−0.58 ± 0.38	−25.0 ± 9.4	0.0016	0.0035	0.676	↓	HMDB0001426	Folate derivatives breakdown, protein and nucleic acid demethylations, glycine and serine metabolisms [13,14]
Hexanal *^e, L1^*	−0.47 ± 0.38	−24.8 ± 11.3	0.0178	0.0245	0.632	↓	HMDB0005994	Aldehyde oxidation (ALDH) and lipid peroxidation [12]
***Aromatic compounds***								
1-Methylnaphthalene *^d, L2^*	0.69 ± 0.39	46.6 ± 10.8	0.0038	0.0070	0.661	↑	HMDB0032860	−
2-Methylnaphthalene *^d, L2^*	0.67 ± 0.39	43.5 ± 10.4	0.0048	0.0083	0.657	↑	−	−
1,2,4-Trimethylbenzene *^d, L2^*	0.57 ± 0.38	43.7 ± 12.3	0.0077	0.0121	0.648	↑	HMDB0013733	−
*p*-Cresol (4-methylphenol) *^d, L1^*	0.61 ± 0.38	136.3 ± 26.4	0.0008	0.0021	0.686	↑	HMDB0001858	Tyrosine and phenylalanine metabolism [15]
***Heterocyclic compounds***								
(1S,5R)-1,5-dimethyl-6,8-dioxabicyclo[3.2.1]octane *^d, L2^*	−0.48 ± 0.38	−34.4 ± 16.5	0.0018	0.0037	0.674	↓	−	−
*Ketones*								
2-Butanone (butan-2-one) *^e, L1^*	−0.54 ± 0.38	−23.8 ± 9.5	0.0004	0.0012	0.697	↓	HMDB0000474	Fatty acid metabolism (*β*-oxidation) [12]
4-Heptanone (heptan-4-one) *^e, L1^*	−0.33 ± 0.38	−38.6 ± 27.7	0.0003	0.0010	0.703	↓	HMDB0004814	Fatty acid metabolism (*β*-oxidation) [12]
***Terpenoids***								
Carvone (2-methyl-5-(prop-1-en-2-yl)cyclohex-2-en-1-one) *^d, L1^*	−0.66 ± 0.39	−62.3 ± 25.5	0.0001	0.0004	0.715	↓	HMDB0035824	Lipid and carbohydrate metabolisms [16]
Piperitone (3-methyl-6-propan-2-ylcyclohex-2-en-1-one) *^d, L2^*	−0.44 ± 0.38	−57.5 ± 34.1	0.0019	0.0037	0.673	↓	HMDB0034975	Lipid metabolism [17]
***Unknowns***								
Unknown 1 *^d, L4^*	0.45 ± 0.38	75.0 ± 23.5	0.0157	0.0225	0.634	↑	−	−
Unknown 2 *^d, L4^*	0.72 ± 0.39	153.2 ± 24.0	<0.0001	0.0003	0.726	↑	−	−
Unknown 3 *^d, L4^*	0.63 ± 0.38	68.1 ± 16.0	0.0061	0.0101	0.653	↑	−	−
Unknown 4 *^d, L4^*	0.41 ± 0.38	269.6 ± 55.8	0.0001	0.0003	0.725	↑	−	−
Unknown 5 *^d, L4^*	−0.50 ± 0.38	−58.2 ± 30.8	0.0003	0.0010	0.702	↓	−	−
Unknown 6 *^e, L4^*	−0.46 ± 0.38	−23.9 ± 11.2	0.0303	0.0383	0.621	↓	−	−

*^a^* Common metabolite name (IUPAC name). *^b^* Effect size ± ES_SE_ (effect size standard error) determined as described in reference [18]. *^c^*False discovery rate (FDR) correction of original *p*-values computed as described in reference [19]. *^d e^* Compounds detected through VOCs and VCCs analytical methods, respectively. Levels of confidence in metabolite identification, defined as described in references [20,21]: *^L1^* Identified metabolites (confirmed using a chemical reference standard); *^L2^* Putatively annotated compounds (NIST14 database); *^L3^* Putatively characterized compound classes (spectral MS similarity); *^L4^* Unknown compounds.

**Table 3:** Performance of the set of seventeen significantly different volatiles for detection of BC stages by ROC analysis.

Groups Compared	AUC	Sensitivity	Specificity	Accuracy
Stage Ta/Tis (*n* = 26) vs. controls (*n* = 56)	0.761	65%	84%	78%
Stage T1 (*n* = 17) vs. controls (*n* = 56)	0.910	94%	80%	84%
Stages ≥ T2 (*n* = 10) vs. controls (*n* = 56)	0.820	60%	91%	86%

## Data Availability

Data available on request due to privacy restrictions.

## Supplementary Materials

The following are available online at https://www.mdpi.com/article/10.3390/metabo11040199/s1, Figure S1: PCA scores scatter plots obtained for urinary volatile profile (full set of VOCs and VCCs concatenated matrix) of all samples (BC *n* = 53 and cancer-free controls *n* = 56, green circles) and QCs (*n* = 24, blue circles), Figure S2: Statistical validation of the PLS-DA model obtained for the urinary volatile profile of BC patients (*n* = 53) and cancer-free controls (*n* = 56), after variable selection (109 observations × 4184 variables), by permutation testing (200 permutations; 2 components), Figure S3: (a) PLS-DA scores scatter plot and (b) ROC curve obtained for urinary volatile profile (VOCs and VCCs concatenated matrix), after variable selection (43 observations × 495 variables), of stage T1 (red squares, *n* = 17) compared with stage Ta/Tis (blue circles, *n* = 26). (c) Statistical validation of the PLS-DA model by permutation testing (500 permutations; 2 components), Figure S4: Statistical validation of the PLS-DA model obtained for urinary volatile profile of (a) patients diagnosed with MIBC (stages ≥ T2, *n* = 10) and NMIBC (stage Ta/Tis, *n* = 26), after variable selection (36 observations × 486 variables), and (b) patients diagnosed with MIBC (stages ≥T2, *n* = 10) and NMIBC (stage T1, *n* = 17), after variable selection (27 observations × 856 variables), by permutation testing (200 permutations; 2 components), Table S1: List of volatile compounds significantly altered in urine of BC patients compared to cancer-free controls and between different BC stages, including their retention time (RT), most abundant ions, NIST and experimental retention index (RI), R-match, CAS number and identification level, Table S2: Correlation coefficients and corresponding *p*-values computed between age and the set of metabolites found altered in urine of BC (*n* = 53) compared to cancer-free controls (*n* = 56), Table S3: List of five volatile metabolites found altered in urine of patients diagnosed with stage T1 (*n* = 17) compared with stage Ta/Tis (*n* = 26), Table S4: List of eight volatile metabolites found altered in urine of patients diagnosed with stages ≥T2 (n = 10) compared with stage Ta/Tis (*n* = 26), Table S5: List of six volatile metabolites found altered in urine of patients diagnosed with stages ≥T2 (*n* = 10) compared with stage T1 (*n* = 17), Table S6: Pre-processing steps of GC-MS chromatograms of VOCs and VCCs performed in MZmine-2.52.
