# Supplementary material for: Discovery of Volatile Biomarkers for Bladder Cancer Detection and Staging through Urine Metabolomics
Source: Metabolites. 2021 Mar 26;11(4):199. doi: 10.3390/metabo11040199 (PMC8066175; doi:10.3390/metabo11040199)
Supplement: Supplementary file 1 [file metabolites-11-00199-s001.pdf]

Supporting Information

Discovery of volatile biomarkers for bladder cancer detection and staging through urine metabolomics

Joana Pinto [1,*], Ângela Carapito [1], Filipa Amaro [1], Ana Rita Lima [1], Carina Carvalho-Maia [2,3], Maria Conceição Martins [2,3], Carmen Jerónimo [1,2,3], Rui Henrique [1,2,3], Maria de Lourdes Bastos [1] and Paula Guedes de Pinho [1,*]

[1] UCIBIO/REQUIMTE, Department of Biological Sciences, Laboratory of Toxicology, Faculty of Pharmacy, University of Porto, 4050-313, Porto, Portugal

[2] Cancer Biology & Epigenetics Group–Research Centre, Portuguese Oncology Institute of Porto (CI-IPOP), 4200-072, Porto, Portugal

[3] Department of Pathology, Portuguese Oncology Institute of Porto, 4200-072, Porto, Portugal

[4] Department of Pathology and Molecular Immunology, Institute of Biomedical Sciences Abel Salazar–University of Porto (ICBAS-UP), 4050-313, Porto, Portugal

*Corresponding authors

E-mail addresses:

jipinto@ff.up.pt (Joana Pinto)

pguedes@ff.up.pt (Paula Guedes de Pinho)

Address:

UCIBIO/REQUIMTE, Laboratory of Toxicology

Department of Biological Sciences

Faculty of Pharmacy

University of Porto

Rua Jorge Viterbo Ferreira, 228

4050-313 Porto, Portugal

Tel.: +351 220428599; fax: +351 226093390

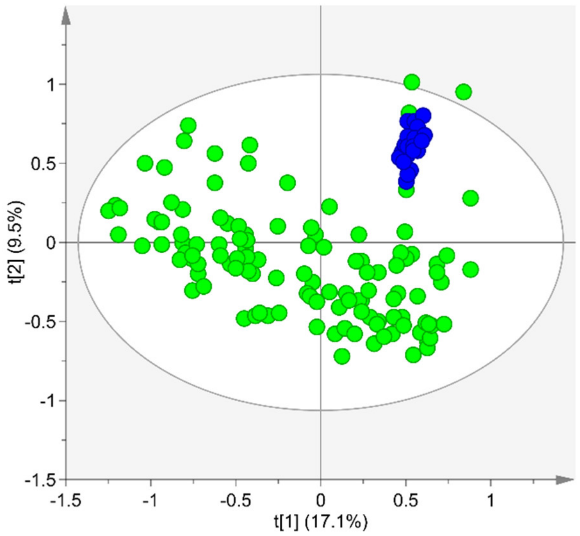

Figure S1. PCA scores scatter plots obtained for urinary volatile profile (full set of VOCs and VCCs concatenated matrix) of all samples (BC *n*=53 and cancer-free controls *n*=56, green circles) and QCs (*n*=24, blue circles).

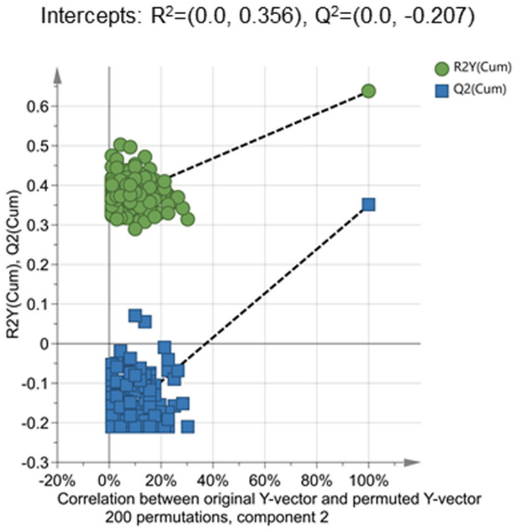

Figure S2. Statistical validation of the PLS-DA model obtained for the urinary volatile profile of BC patients (*n*=53) and cancer-free controls (*n*=56), after variable selection (109 observations × 4184 variables), by permutation testing (200 permutations; 2 components).

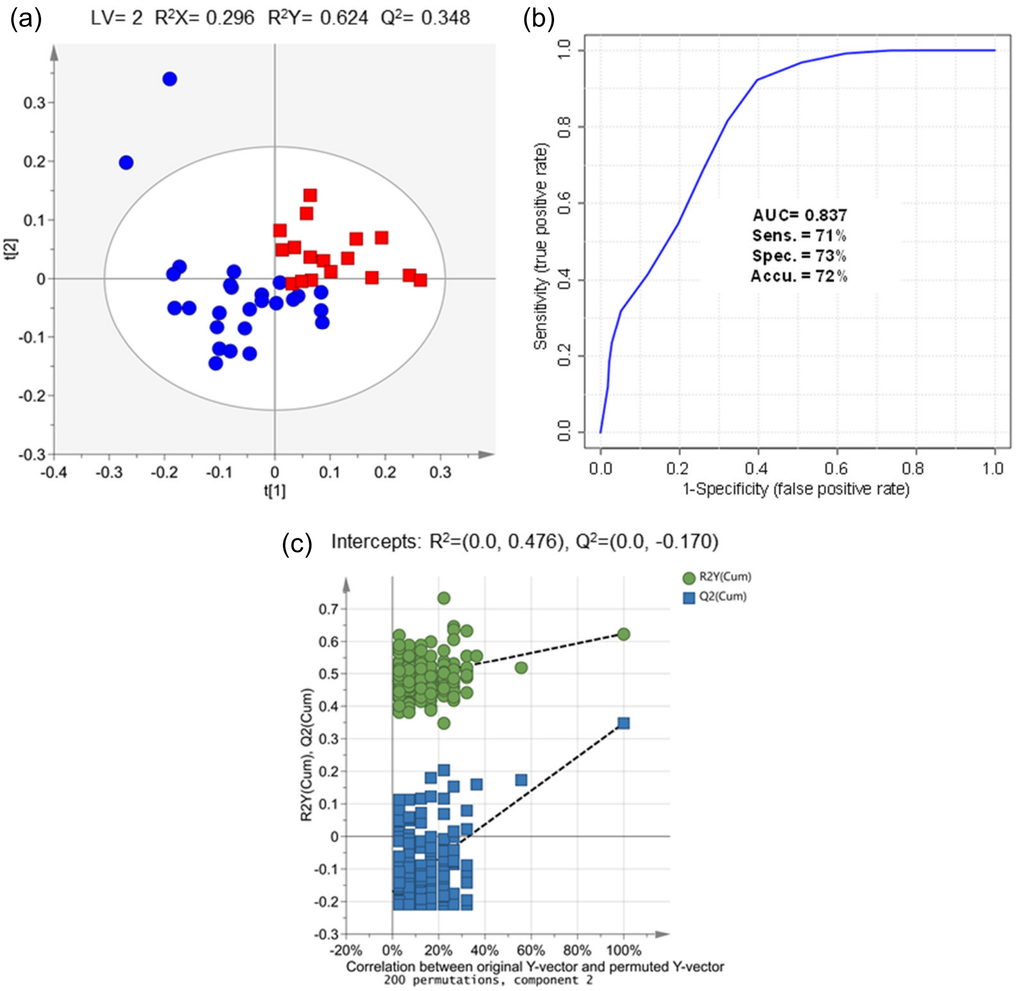

Figure S3. (a) PLS-DA scores scatter plot and (b) ROC curve obtained for urinary volatile profile (VOCs and VCCs concatenated matrix), after variable selection (43 observations × 495 variables), of stage T1 (red squares, *n*=17) compared with stage Ta/Tis (blue circles, *n*=26). (c) Statistical validation of the PLS-DA model by permutation testing (500 permutations; 2 components).

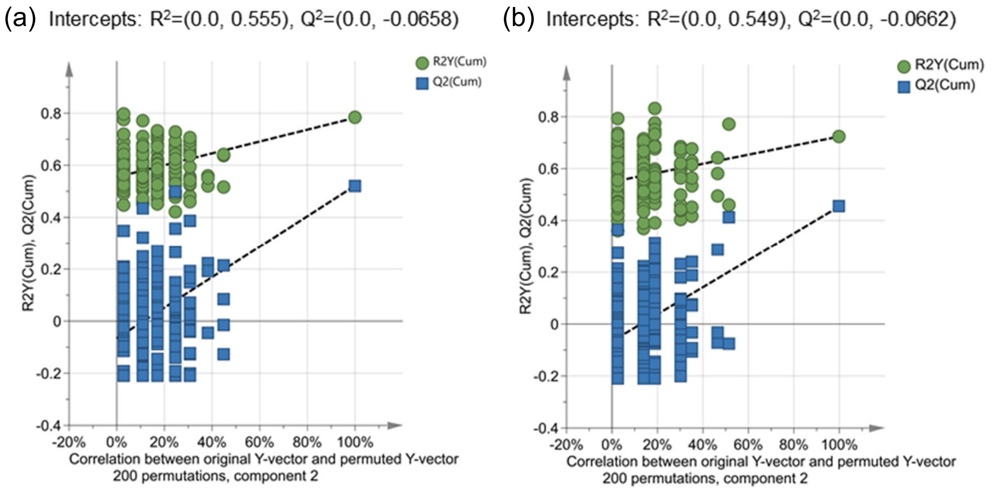

Figure S4. Statistical validation of the PLS-DA model obtained for urinary volatile profile of (a) patients diagnosed with MIBC (stages ≥T2, *n*=10) and NMIBC (stage Ta/Tis, *n*=26), after variable selection (36 observations × 486 variables), and (b) patients diagnosed with MIBC (stages ≥T2, *n*=10) and NMIBC (stage T1, *n*=17), after variable selection (27 observations × 856 variables), by permutation testing (200 permutations; 2 components).

Table S1. List of volatile compounds significantly altered in urine of BC patients compared to cancer-free controls and between different BC stages, including their retention time (RT), most abundant ions, NIST and experimental retention index (RI), R-match, CAS number and identification level.

Metabolite [a]	RT (min)	Characteristic ions (*m/z*)	NIST RI [b]	Experimental RI [c]	R-match	CAS number	Identification level [d]
Alkanes							
2-Methylnonane [e]	9.46	57/71/85	964	966	776	871-83-0	L2
2,4-Dimethylheptane [e]	5.47	57/71/85	821	823	908	2213-23-2	L2
2,6-Dimethylnonane [e]	11.15	57/71/85	1018	1022	881	17302-28-2	L2
4-Methyloctane [e]	6.53	57/71/85	863	863	907	2216-34-4	L2
Decane [e]	10.50	57/71/85	1000	1001	831	124-18-5	L1
Aldehydes							
2-Furaldehyde (furan-2-carbaldehyde) [f]	23.16/23.63	83/181/248/291	-	1464/1483	965	98-01-1	L1
2-Methylbutanal [e]	2.80	57/58/86	662	-	842	96-17-3	L1
2,5-Dimethylbenzaldehyde [e]	16.85	77/91/105/133	1208	1213	915	5779-94-2	L2
Formaldehyde [f]	9.92	117/161/181/195	-	995	874	50-00-0	L1
Hexanal [f]	22.36	181/239	-	1433	836	66-25-1	L1
Methylglyoxal [f]	33.02/33.21/33.52	181/265	-	1907/1917/1933	943	78-98-8	L1
Octanal [e]	10.56	57/69/84	1003	1003	811	124-13-0	L1
Aromatic hydrocarbons							
1-Methylnaphthalene [e]	19.53	71/115/141/142	1307	1310	854	90-12-0	L2
1,2,3-Trimethylbenzene [e]	11.08	91/105/120	1013	1020	873	526-73-8	L2
1,2,4-Trimethylbenzene [e]	10.31	77/91/105/120	990	994	912	95-63-6	L2
1,2,4,5-Tetramethylbenzene [e]	14.06	91/119/134	1116	1118	877	95-93-2	L2

Metabolite [a]	RT (min)	Characteristic ions (*m/z*)	NIST RI [b]	Experimental RI [c]	R-match	CAS number	Identification level [d]
2-Methylnaphthalene [e]	19.11	71/115/141/142	1298	1294	868	91-57-6	L2
2-Ethyl-1,3-dimethylbenzene [e]	13.00	91/119/134	1080	1083	904	2870-04-4	L2
p-Cresol (4-methylphenol) [e]	12.65	77/107/108	1077	1071	908	106-44-5	L1
Heterocyclic Compounds							
(1S,5R)-1,5-dimethyl-6,8-dioxabicyclo[3.2.1]octane [e]	8.73	72/100/142	949	942	824	28401-39-0	L2
Ketones							
2-Butanone (butan-2-one) [f]	16.62/16.71	56/181/195/250	-	1219/1222	836	78-93-3	L1
4-Heptanone (heptan-4-one) [f]	22.02	70/128/181/253/309	-	1419	867	123-19-3	L1
Terpenoids							
Carvone (2-methyl-5-(prop-1-en-2-yl)cyclohex-2-en-1-one) [e]	17.67	54/82/93/108	1242	1243	871	99-49-0	L1
Levomenthol ((1R,2S,5R)-5-methyl-2-propan-2-ylcyclohexan-1-ol) [e]	15.79	71/81/95/123/138	1175	1190	946	2216-51-5	L2
Piperitone (3-methyl-6-propan-2-ylcyclohex-2-en-1-one) [e]	17.93	82/95/110/137/152	1253	1252	889	89-81-6	L2
Unknowns							
Unknown 1 [e]	12.00	68/105/116	-	1050	-	-	L4
Unknown 2 [e]	12.16	57/71/85	-	1055	-	-	L4
Unknown 3 [e]	12.26	57/71/85	-	1062	-	-	L4
Unknown 4 [e]	16.58	135/164	-	1204	-	-	L4

Metabolite [a]	RT (min)	Characteristic ions (*m/z*)	NIST RI [b]	Experimental RI [c]	R-match	CAS number	Identification level [d]
Unknown 5 [e]	18.47	137/152	-	1271	-	-	L4
Unknown 6 [f]	19.61	181/226	-	1327	-	-	L4
Unknown 7 [e]	14.15	79/91	-	1121	-	-	L4
Unknown 8 [e]	15.24	83/121/136	-	1158	-	-	L4
Unknown 9 [f]	23.52	161/181	-	1479	-	-	L4

[a] Common metabolite name (IUPAC name). [b] Theoretical Kovats retention index (RI) extracted from NIST 14 database. [c] Experimental Kovats RI determined using a commercial hydrocarbon mixture (C6–C20). [d] Levels of confidence in metabolite identification [21,22]: L1 - identified metabolites (GC-MS analysis of the metabolite of interest and a chemical reference standard under identical analytical conditions within the same laboratory); L2 - putatively annotated compounds (spectral MS similarity with the NIST database); L3 - putatively characterized compound classes (spectral MS consistent with a particular class of organic compounds); L4 - unknown compounds. [e, f] Compounds detected through VOCs and VCCs analytical methods, respectively.

Table S2. Correlation coefficients and corresponding *p*-values computed between age and the set of metabolites found altered in urine of BC (n=53) compared to cancer-free controls (n=56).

Metabolite [a]	Correlation coefficient (r)	p-value
Alkanes		
2-Methylnonane	0.23	0.0160
2,4-Dimethylheptane	0.23	0.0159
2,6-Dimethylnonane	0.28	0.0027
4-Methyloctane	0.26	0.0074
Aldehydes		
2-Furaldehyde (furan-2-carbaldehyde)	-0.16	0.1062
2-Methylbutanal	-0.23	0.0172
Formaldehyde	-0.21	0.0252
Glyoxal	-0.10	0.3068
Hexanal	-0.16	0.0909
Aromatic hydrocarbons		
1-Methylnaphthalene	0.31	0.0009
2-Methylnaphthalene	0.27	0.0041
1,2,4-Trimethylbenzene	0.29	0.0020
p-Cresol (4-methylphenol)	0.22	0.0203
Heterocyclic Compounds		
(1S,5R)-1,5-dimethyl-6,8-dioxabicyclo[3.2.1]octane	-0.31	0.0012
Ketones		
2-Butanone (butan-2-one)	-0.25	0.0079
4-Heptanone (heptan-4-one)	-0.12	0.2125
Terpenoids		
Carvone (2-methyl-5-(prop-1-en-2-yl)cyclohex-2-en-1-one)	-0.31	0.0012
Piperitone (3-methyl-6-propan-2-ylcyclohex-2-en-1-one)	-0.28	0.0032
Unknowns		
Unknown 1	0.19	0.0457
Unknown 2	0.24	0.0112
Unknown 3	0.18	0.0674
Unknown 4	0.16	0.0898
Unknown 5	-0.20	0.0001
Unknown 6	-0.28	0.0029

Table S3. List of five volatile metabolites found altered in urine of patients diagnosed with stage T1 (*n*= 17) compared with stage Ta/Tis (*n*= 26).

Metabolite [a]	Effect size ± ES_{SE} [b]	Variation ± uncertainty (%)	*p*-value original	*p*-value FDR [c]	AUC	Down- or up-regulated	HMDB ID	Potential biochemical pathway
Alkane								
Decane [d, 12]	0.63 ± 0.48	183.7 ± 21.3	0.0475	0.0633	0.681	↑	HMDB0031450	Lipid peroxidation [12]
Aldehyde								
Octanal [d, L1]	0.72 ± 0.48	66.7 ± 12.1	0.0294	0.0632	0.674	↑	HMDB0001140	Lipid peroxidation [12]
Terpenoid								
Levomenthol ((1R,2S,5R)-5-methyl-2-propan-2-ylcyclohexan-1-ol) [d, L2]	-0.76 ± 0.48	-57.0 ± 34.5	0.0177	0.0632	0.715	↓	HMDB0003352	Lipid metabolism [17]
Unknowns								
Unknown 7 [d, L4]	0.36 ± 0.47	139.2 ± 32.2	0.0371	0.0632	0.690	↑	-	-
Unknown 8 [d, L4]	-0.96 ± 0.49	-63.4 ± 37.9	0.0040	0.0320	0.758	↓	-	-

[a] Common metabolite name (IUPAC name). [b] Effect size ± ES_{SE} (effect size standard error) determined as described in reference [21]. [c] False discovery rate (FDR) correction of original *p*-values, computed as described in reference [20]. [d, e] Compounds detected through VOCs and VCCs analytical methods, respectively. Levels of confidence in metabolite identification, defined as described in references [16,17]: [L1] Identified metabolites (confirmed using a chemical reference standard); [L2] Putatively annotated compounds (NIST14 database); [L3] Putatively characterized compound classes (spectral MS similarity); [L4] Unknown compounds.

Table S4. List of eight volatile metabolites found altered in urine of patients diagnosed with stages ≥T2 (*n*= 10) compared with stage Ta/Tis (*n*=26).

**Metabolite** [a]	**Effect size ± ES_{SE}** [b]	**Variation ± uncertainty (%)**	***p*-value original**	***p*-value FDR** [c]	**AUC**	**Down- or up-regulated**	**HMDB ID**	**Potential biochemical pathway**
Alkanes								
2,4-Dimethylheptane [d, L2]	1.86 ± 0.85	260.1 ± 46.3	0.0002	0.0010	0.881	↑	-	-
4-Methyloctane [d, L2]	1.49 ± 0.81	493.9 ± 81.2	0.0001	0.0004	0.904	↑	-	-
Decane [d, L1]	1.39 ± 0.79	186.1 ± 52.5	0.0055	0.0078	0.796	↑	HMDB0031450	Lipid peroxidation [12]
Aldehydes								
2,5-Dimethylbenzaldehyde [d, L2]	-0.81 ± 0.75	-31.2 ± 10.7	0.0037	0.0071	0.692	↓	HMDB0032014	-
Aromatic compounds								
1,2,3-Trimethylbenzene [d, L2]	0.72 ± 0.75	50.6 ± 22.4	0.0310	0.0344	0.735	↑	HMDB0059901	-
1,2,4-Trimethylbenzene [d, L2]	0.77 ± 0.75	45.7 ± 19.2	0.0281	0.0344	0.738	↑	HMDB0013733	-
1,2,4,5-Tetramethylbenzene [d, L2]	1.22 ± 0.78	97.5 ± 24.6	0.0042	0.0071	0.804	↑	-	-
Unknowns								
Unknown 2 [d, L4]	1.34 ± 0.79	228.6 ± 59.4	0.0025	0.0071	0.819	↑	-	

[a] Common metabolite name (IUPAC name). [b] Effect size ± ES_{SE} (effect size standard error) determined as described in reference [21]. [c] False discovery rate (FDR) correction of original *p*-values, computed as described in reference [20]. [d, e] Compounds detected through VOCs and VCCs analytical methods, respectively. Levels of confidence in metabolite identification, defined as described in references [16,17]: [L1] Identified metabolites (confirmed using a chemical reference standard); [L2] Putatively annotated compounds (NIST14 database); [L3] Putatively characterized compound classes (spectral MS similarity); [L4] Unknown compounds.

Table S5. List of six volatile metabolites found altered in urine of patients diagnosed with stages ≥T2 (*n*= 10) compared with stage T1 (*n*=17).

Metabolite [a]	Effect size ± ES_{SE} [b]	Variation ± uncertainty (%)	*p*-value original	*p*-value FDR [c]	AUC	Down- or up-regulated	HMDB ID	Potential biochemical pathway
Alkane								
2,4-Dimethylheptane [d, L2]	1.06 ± 0.83	138.8 ± 38.0	0.0039	0.0165	0.829	↑	-	-
Aldehydes								
2,5-Dimethylbenzaldehyde [d, L2]	-0.88 ± 0.82	-21.0 ± 9.0	0.0419	0.0419	0.747	↓	HMDB0032014	-
Formaldehyde [f, L1]	0.90 ± 0.82	81.0 ± 30.7	0.0401	0.0419	0.724	↑	HMDB0001426	Folate derivatives breakdown, protein and nucleic acid demethylations, glycine and serine metabolisms [13,14]
Methylglyoxal [f, L1]	1.10 ± 0.83	58.4 ± 19.2	0.0141	0.0247	0.776	↑	HMDB0001167	Pyruvate metabolism, glycine and serine metabolism, spermidine and spermine biosynthesis [17]
Terpenoid								
Levomenthol ((1R,2S,5R)-5-methyl-2-propan-2-ylcyclohexan-1-ol) [d, L2]	1.07 ± 0.83	207.7 ± 50.4	0.0047	0.0165	0.824	↑	HMDB0003352	-
Unknowns								
Unknown 9 [d, L4]	1.17 ± 0.84	129.2 ± 37.3	0.0093	0.0218	0.800	↑	-	-

[a] Common metabolite name (IUPAC name). [b] Effect size ± ES_{SE} (effect size standard error) determined as described in reference [21]. [c] False discovery rate (FDR) correction of original *p*-values, computed as described in reference [20]. [d, e] Compounds detected through VOCs and VCCs analytical methods, respectively. Levels of confidence in metabolite identification, defined as described in references [16,17]: [L1] Identified metabolites (confirmed using a chemical reference standard); [L2] Putatively annotated compounds (NIST14 database); [L3] Putatively characterized compound classes (spectral MS similarity); [L4] Unknown compounds.

Table S6. Pre-processing steps of GC-MS chromatograms of VOCs and VCCs performed in MZmine-2.52.

Pre-processing steps	VOCs	VCCs
Crop filtering	RT range: 2-34 min<br>*m/z* range: 50-250	RT range: 9.8-46 min<br>*m/z* range: 50-300
Peak detection	Noise level: 1×10^4	Noise level: 1×10^5
Chromatogram builder	Intensity threshold: 5×10^4<br>*m/z* tolerance: 0.07	Intensity threshold: 5×10^6<br>*m/z* tolerance: 0.1
Deconvolution	Baseline level: 1×10^4<br>Peak range: 0.03-0.5 min	Baseline level: 5×10^5<br>Peak range: 0.03-0.5 min
Alignment	RT tolerance: 0.2 min<br>*m/z* tolerance: 0.07	RT tolerance: 0.2 min<br>*m/z* tolerance: 0.1
